# Retraction Note: A positive feedback loop consisting of C12orf59/NF-κB/CDH11 promotes gastric cancer invasion and metastasis

**DOI:** 10.1186/s13046-021-02171-7

**Published:** 2021-11-12

**Authors:** Zhang Jia-Xing, He Wei-Ling, Feng Zi-Hao, Chen Dong-Liang, Gao Ying, He Ying, Qin Kai, Zheng Zhou-San, Chen Cui, Weng Hui-Wen, Yun Miao, Ye Sheng, Xu Rui-Hua, Xie Dan

**Affiliations:** 1 The State Key Laboratory of Oncology in South China, Sun Yat-Sen University Cancer Center, Collaborative Innovation Center for Cancer Medicine, No. 651, Dongfeng Road East, 510060 Guangzhou, Guangdong Province People’s Republic of China; 2 Department of Oncology, the First Affiliated Hospital, Sun Yat-Sen University, Guangzhou, 510080 Guangdong Province People’s Republic of China; 3 Department of Gastrointestinal Surgery, The First Affiliated Hospital, Sun Yat-Sen University, Guangzhou, 510080 Guangdong Province People’s Republic of China; 4 Department of Urology, the First Affiliated Hospital, Sun Yat-Sen University, Guangzhou, 510080 Guangdong Province People’s Republic of China; 5 Department of Extracorporeal Circulation, the First Affiliated Hospital, Sun Yat-Sen University, Guangzhou, 510080 Guangdong Province People’s Republic of China

**Retraction Note: J Exp Clin Cancer Res 38, 164 (2019)**

**https://doi.org/10.1186/s13046-019-1114-2**

The Editor-in-Chief has retracted this article after the authors alerted the journal to issues in a number of the figures. Specifically, the affected figures are:

- Fig. 2d: Migration image of shC12orf59#1 on MKN-45 cells (upper middle); Migration and Invasion images of shC12orf59 on AGS cells (right)
- Fig. 2e: Invasion images of vector and C12orf59 on HGC-27 cells (lower)
- Fig. 4e: Invasion image of shControl on MKN-45 cells (lower left)
- Fig. 4i: Migration image of shC12orf59#1 on HUVEC cells (middle)
- Fig. 5h: Invasion image of C12orf59 on MKN-45 cells (lower middle)
- Fig. S6b: Migration and Invasion images of miR-654-5p and control on MNK-45 and AGS cells

The Editor-in-Chief therefore no longer has confidence in the results and conclusions presented.

Authors Zhang Jia-Xing, Zheng Zhou-San, Ye Sheng, Xu Rui-Hua & Xie Dan agree to this retraction. Authors He Wei-Ling, Feng Zi-Hao, Chen Dong-Liang, Gao Ying, He Ying, Qin Kai, Chen Cui, Weng Hui-Wen, Yun Miao have not responded to any correspondence from the editor or publisher about this retraction.

